# Coping with and self-management of chronic painful chemotherapy-induced peripheral neuropathy: a qualitative study among cancer survivors

**DOI:** 10.1007/s11764-023-01466-2

**Published:** 2023-09-23

**Authors:** Daniëlle L. van de Graaf, Floortje Mols, Tom Smeets, Hester R. Trompetter, Marije L. van der Lee

**Affiliations:** 1 Center of Research on Psychological disorders and Somatic diseases (CoRPS), Department of Medical and Clinical Psychology, Tilburg University, PO Box 90153, 5000 LE Tilburg, The Netherlands; 2 Department of Research, Netherlands Comprehensive Cancer Organisation (IKNL), Utrecht, The Netherlands; 3 Centre for Psycho-Oncology, Scientific Research Department, Helen Dowling Institute, Bilthoven, The Netherlands

**Keywords:** CIPN, Coping, Self-management, Quality of life

## Abstract

**Purpose:**

Patients with chronic painful chemotherapy-induced peripheral neuropathy (CIPN) may experience a negative impact of CIPN on daily life. They can use various coping (i.e., dealing with symptoms and resulting impairments in general) and self-management (i.e., practical actions to reduce symptoms) strategies to live with their limitations. This paper aimed to examine experienced helpful coping and self-management strategies of patients with chronic painful CIPN.

**Methods:**

Semi-structured interviews were conducted with twelve patients with chronic painful CIPN. We applied a hybrid deductive-inductive coding approach. ATLAS.ti was used for coding.

**Results:**

Generated from the data were two themes and nine codes for coping and four themes and 31 codes for self-management strategies. Coping of patients often included active strategies like planning, seeking social support, and acceptance. Additionally, patients often used passive strategies such as focusing on and venting emotions and suppressing competing activities. The most common self-management strategies were mostly passive (i.e., medication, deliberate choice of shoes, resting, sitting, and consulting healthcare professionals) but also active (i.e., exercising) strategies.

**Conclusion:**

Patients exhibit a great variety of coping and self-management strategies that they perceive as helpful to deal with chronic painful CIPN. However, research has shown that certain strategies are not that helpful or even come with aversive effects. More research into the effectiveness and implementation of psychosocial interventions is needed since it may help patients adopting helping strategies. In addition, healthcare professionals need to refer patients with CIPN in a timely manner to physical therapists, occupational therapists, or rehabilitation teams to reduce or prevent (further) impairments.

**Implications for Cancer Survivors:**

Patients can consult one of their healthcare providers in case of problems in dealing with their symptoms, to get proper guidance and possible referral.

## Introduction

A large proportion of cancer survivors suffer from long-term effects of their diagnosis and treatment, such as chemotherapy-induced peripheral neuropathy (CIPN) [1]. This condition is caused by certain chemotherapeutic agents, such as taxanes, platinum compounds, and vinca alkaloids [2–4]. After completion of chemotherapy, around 80% of patients suffer from this, decreasing to around 30% at 6 months or later [1, 5–9]. CIPN presents itself in the hands and feet, but can also spread to arms and legs [10–12]. Symptoms that patients experience greatly vary, which is influenced by chemotherapy type and cumulative dose, as well as patient characteristics and perceptions [13]. Symptoms may include non-painful symptoms, such as tingling or numbness, as well as painful symptoms, such as shooting or burning pain, pins-and-needles, and cramps [10–12, 14].

Currently, pharmacological treatment options for CIPN are limited [15]. Non-pharmacological treatment options for patients with CIPN are currently increasingly examined in international studies and could be promising [16–19]. To date, however, there are no general evidence-based non-pharmacological treatment options offered to chronic painful CIPN patients [15, 20, 21]. Since the symptoms patients experience strongly vary, the experienced limitations are also diverse. Some patients experience difficulty with even minor daily activities, such as buttoning up a blouse or holding a pen [22]. Consequences range from limitations in activities of daily living (ADL), such as walking, sleeping, and intimacy/sexuality, to limitations in social and role activities, such as taking care of (grand)children, working, and sports [22–24], which may complicate or disable participating in daily life. This can eventually result in deteriorated mood, depressive symptoms, performance of regular activities, and quality of life (QoL) [13, 25–29]. Thereby, previous research has shown that in QoL, patients with painful CIPN present worse outcomes than patients with non-painful CIPN [14].

Patients with chronic painful CIPN need to learn to live with the symptoms and limitations and thus how to cope with it. Coping includes performing a response to a perception of stress and relates to cognitive and behavioral actions to manage internal and external stressors [30, 31]. Psychological stress is defined as “a particular relationship between the person and the environment that is appraised by the person as exceeding his or her resources and endangering well-being” (Lazarus & Folkman, 1984, p. 19). Patients with chronic painful CIPN therefore frequently or continuously have a stressor (i.e., painful neuropathic sensations and subsequent limitations) in their daily lives to which they may apply various coping strategies. This involves both active coping, such as planning and acceptance, and passive coping, such as focusing on and venting emotions, restraint coping (i.e., not acting prematurely and taking action only when the stressor occurs), and suppressing competing activities (e.g., setting aside activities to avoid the stressor) [32]. However, the usefulness of these coping strategies varies widely, as certain strategies (e.g., behavioral disengagement and mental disengagement) are less useful [32].

Patients may also take practical actions to be able to perform activities of daily living despite chronic painful CIPN. This is referred to as self-management strategies, where the aim is to relieve symptoms [33]. More specifically, it includes patients performing activities or directing others to perform activities to remove or reduce symptoms and associated stress and is also known as symptom management [34]. Self-management strategies can be divided into two categories [33]. Active strategies include activities initiated by the patient to deal with the pain, but do not involve avoidance or escape behavior. This can include both active (e.g., exercise or modified use) and cognitive (e.g., relaxation or distraction) strategies. Passive strategies mean that treatment was offered or provided, without the patient having to actively contribute, or that the patient allows other aspects of their lives to be negatively affected by pain. It may include both passive behavior (e.g., rest or drinking alcohol) and conventional medical strategies (e.g., medication or physiotherapist). Here, avoidance and escape behavior do apply. Passive strategies often have aversive long-term effects, since these may eventually result in impaired functioning [35]. Reduced functioning may relate to emotional, cognitive, social, role, and physical aspects (e.g., emotional distress, pain, sleep problems, loss of purpose) [13, 14, 23, 36].

Conversely, active self-management strategies contribute to reduced pain-related disability, distress, medication, and pain-related healthcare visits [33].

Knowledge of the helping active as well as aversive passive strategies that people employ could contribute to the development of appropriate interventions that encourage patients to adopt active coping and self-management strategies, to enhance health-related outcomes. To our knowledge, no previous research studied both coping and self-management strategies of patients with chronic painful CIPN. Since self-management strategies are practical ways of dealing with symptoms and thus part of coping, it is important to look at both within one sample. Therefore, the aim of this paper was to examine coping and self-management strategies that are perceived as helpful by patients with chronic painful CIPN. In this study, we consider coping as dealing with the stressor (i.e., painful neuropathic sensations and resulting impairments) in general and self-management as a component of coping in which patients take practical actions to reduce symptoms.

## Method

### Participants

This study was approved by the Ethical Review Board of Tilburg University (School of Social and Behavioral Sciences; #RP284). Participants had to (1) be 18 years or older, (2) experience chronic painful CIPN for at least 3 months, (3) experience self-reported interference of chronic painful CIPN with daily life activities, (4) be in the curative disease phase, and (5) score 3 or higher on an 11-point Numeric Rating Scale (NRS) to assess pain severity. Exclusion criteria were receiving chemotherapy or psychosocial treatment at the time of inclusion. Inclusion and exclusion criteria were based on earlier articles that were conducted in similar research projects [17, 37].

Participants were recruited via online recruitment flyers via patient organizations and *Kanker.nl* (i.e., Dutch unified web platform delivering tailored medical information and peer-support for cancer patients and relatives [38]). Patients could apply by sending an e-mail to the interviewer (DG). An information letter and informed consent form were sent to interested patients. If the informed consent was filled out and returned, an interview was scheduled. No financial incentives were provided to participants.

### Data collection and procedures

Semi-structured interviews were conducted by one of the authors (DG) between September and December 2020. Due to COVID-19, interviews took place via video calling. The interview schedule was mainly based on previous qualitative studies on (cancer) patient experiences [39–41]. After compiling a first version of the interview schedule, it was discussed with researchers with expertise in painful and non-painful CIPN (FM) and in (coping with) chronic pain (HT) to check whether essential components to assess dealing with chronic painful CIPN were missing. The final semi-structured interview scheme is shown in Table 1. The interviews mainly focused on the helping strategies in patients’ perceptions. Interviews were conducted until saturation was reached. This resulted in interviews with twelve patients.

**Table 1:** Semi-structured interview scheme

Category	Main questions	Optional follow-up questions
Coping	How do you deal with the symptoms daily?	Are you sometimes afraid to move, to do certain actions or to perform a certain activity, because you think, for example, that the symptoms will worsen? What do you do in such a situation?
	Are there any activities that you can no longer perform yourself or modified due to the symptoms? I would like to ask you to imagine yourself in such a situation.	What kind of emotions do you experience at such a moment?
		What kind of thoughts do you have at such a moment?
		What do you do at such a moment?
	Have you recently experienced having many symptoms before or during an activity that was very important to you? For example, the birthday of your (grand)child or an important work appointment	What do you do specifically to solve the ‘problem’?
		What did you do then?
		Did you make any adjustments?
		How did you feel before, during, and after the activity?
Self-management	What have you tried to reduce or remedy symptoms yourself?	Did it help in the short term?
		Did it help in the long term?
		What does work well for your symptoms?
		Have you ever used medication (drugstore or pharmacy), exercise, psychotherapy, physical therapy, supplements, etc.?
		Which healthcare professionals have you visited for these symptoms?

### Data analysis

Thematic analysis was used for analyzing the data [42]. Interviews were audio recorded and afterwards transcribed verbatim by two third-year Psychology bachelor’s students and one Psychology master’s student from Tilburg University. Transcriptions were re-read by one author (DG) to verify that transcribing had been done correctly and read by a Psychology master’s student to become familiar with the data. The first three interviews were coded by DG and one Medical Psychology master’s student independently. Differences were discussed afterwards and consensus was reached. The remaining interviews were coded by the Psychology master’s student. Difficulties and ambiguities were discussed in weekly meetings between the student and one of the authors (DG).

A hybrid deductive-inductive coding approach was applied [43]. This means that coding was theory-based, but that in addition there was room for new codes to emerge from the data. In this study, coping, which was considered dealing with the stressor (i.e., painful neuropathic sensations and resulting impairments) in general, was completely coded based on earlier research on coping strategies [32]. Self-management was defined as a component of coping, in which patients take practical actions to reduce symptoms, for which codes were based on earlier research on self-management strategies for chronic pain patients [33]. Self-management coding was mainly theory-based [33], but novel themes and subthemes were allowed to emerge from the data. Chronic painful CIPN-specific self-management strategies were not covered by the deductive codes based on the study investigating chronic pain. Since there was a partial overlap between the deductive codes of coping and self-management [32, 33], some codes were eliminated beforehand. Therefore, it was decided that suppression of competing activities and mental disengagement were only coded for coping. Religion and use of alcohol and smoking were only included in the codes of self-management. Furthermore, coping and self-management strategies were only coded when they were perceived as helpful by patients. Strategies that patients had tried in the past but stopped using because they were perceived as non-helpful were not included in the analyses. Data was only coded when strategies were perceived as helpful by at least one patient, as non-helpful strategies may be not beneficial for anyone. It is important to note that text fragments were sometimes coded as both coping and self-management strategies, as quotes can sometimes be related to both (e.g., asking for a chair at a party is included in the coping strategy “active coping/planning” and the self-management strategy “sitting”).

After all transcripts were coded, the codes were reviewed and, once adjustments were made, finalized. Then axial coding started with a codebook of 36 codes. These codes were written down on paper and were sorted by DG and a Medical Psychology master’s student. Sorting was repeated once to ensure that the categories and themes corresponded the data. This resulted in two categories (i.e., coping and self-management) and six overarching themes. Finally, the student created condensed meaning units for each text fragment with codes, to reflect the essence of each participant’s quote. After writing the condensed meaning units, the researcher rechecked whether the codes were appropriate to the corresponding text fragments to improve validity.

## Results

Twelve patients with chronic painful CIPN participated in individual interviews. Patient characteristics are presented in Table 2. The interviews lasted about 30 min on average.

**Table 2:** Participant characteristics

Participant	Gender	Age^a^	Tumor	Time since CIPN onset^a^	Educational level^b^	Marital status
P1	Male	25–30	Hodgkin lymphoma	1–5 years	Middle	Single
P2	Female	50–55	Breast cancer	5–10 years	High	Married/living together
P3	Female	60–65	Breast cancer	>10 years	High	Divorced
P4	Female	60–65	Breast cancer	5–10 years	High	Divorced
P5	Female	60–65	Breast cancer	5–10 years	High	Divorced
P6	Female	60–65	Lung cancer	1–5 years	High	Divorced
P7	Male	60–65	Hodgkin lymphoma	5–10 years	High	Married/living together
P8	Female	60–65	Colorectal cancer	6 months–1 year	Middle	Married/living together
P9	Male	65–70	Leukemia	5–10 years	Middle	Married/living together
P10	Male	65–70	Multiple myeloma	5–10 years	High	Married/living together
P11	Female	75–80	Lung and breast cancer	>10 years	High	Single
P12	Female	75–80	Colorectal and skin cancer	5–10 years	High	Divorced

^a^Ranges are shown to ensure anonymity

^b^Low, primary or secondary pre-vocational; middle, secondary education or vocational education; high, Bachelor’s degree or higher

Results can be divided into two overarching categories: coping strategies and self-management strategies. Two themes (i.e., passive and active) and nine codes for coping strategies emerged from the data. Additionally, four themes (i.e., active behavioral, cognitive (active), passive behavioral, and conventional medical) and 31 codes emerged for self-management strategies. An overview of all strategies is shown in Table 3.

**Table 3:** Types of coping and self-management strategies used

Category	Theme	Code	No. of times coded	No. of participants
Coping	Active	Active coping/planning	34	11
		Seeking social support for emotional and/or instrumental reasons	10	6
		Acceptance	15	5
	Passive	Focus on and venting of emotions	23	8
		Suppression of competing activities	10	5
		Mental disengagement	7	5
		Restraint coping	6	5
		Behavioral disengagement	4	3
		Denial	3	3
Self-management	Active behavioral	Exercise	10	5
		Modified use	5	4
		Perform actions carefully	2	2
		Walking	2	2
		Work	2	1
		Social activities	1	1
	Cognitive (active)	Diary writing	1	1
		Mindfulness	1	1
	Passive behavioral	Shoes	11	8
		Rest	11	7
		Sitting	8	6
		Keeping warm	9	4
		Bed modifications	5	3
		Gloves	5	2
		Feet/legs in certain position	4	3
		Smoking/alcohol	3	3
		Socks	3	3
		Bath/shower	2	2
		Alternative means of transportation	2	2
	Conventional medical	Medication	14	9
		Physiotherapist	7	5
		Orthotics	2	2
		Supplements	2	2
		Psychologist	2	2
		General practitioner	1	1
		Physician	1	1
		Occupational therapist	1	1
		Osteopath	1	1
		Life art coach	1	1
		Coach	1	1
		Rollator walker	1	1

### Coping: active

The most common active coping strategy was planning, mentioned by almost all patients. Many patients indicated that they consider their symptoms when planning their week and make adjustments in advance: “I plan, of course. I sometimes find that a disadvantage. I have to plan incredibly. I can’t go to an exhibition three days in a row” (P2). Many patients indicate that they conduct activities in an alternative manner: “You can say I can’t do things anymore. You can also say I can’t do things as fast as I used to, but I can do them. I just do it more slowly” (P8). Furthermore, several patients indicated that they often search for options that will allow them to continue performing certain activities: “At parties where there are only bar tables … then I really have a lot of pain. I start wobbling from one leg to the other or I start looking for a chair” (P10).

Seeking social support was also mentioned by several patients. They indicated that they reached out to family, friends, and acquaintances for instrumental reasons: “I have to do it all by myself, but of course: if I need to mop the windows, I sometimes ask my daughter ‘can you help?’” (P1). Several patients also indicated that they seek social support for emotional reasons: “It regularly makes me very sad. Surely that’s an average of once a week she [wife] says. My wife always tries to cheer me up” (P11).

Acceptance was also named as helpful by several patients. Many patients report that they cannot change the situation and have learned to live with it: “I have to learn to live with it, because if I don’t, then I have no life” (P7). Some patients put the situation into perspective concerning having cancer and sometimes even trivialize the situation: “Of course it is inconvenient and annoying but well, I think to myself: ‘if the cancer stays away then I just have to be willing to put up with it.’ And that is very annoying, but yes, there are actually worse things, I think. And you can live with it” (P9). In addition, there were a few patients who indicated they were actively making space for the symptoms and dealing with them: “Recently, I started walking. First a round through the village. That’s 3 kilometers. Then another bit further. … So now I can walk 10 kilometers, but my feet do hurt all those 10 kilometers. I always feel them but it’s not like it hurts so much that I can’t walk with them” (P10).

### Coping: passive

The most common passive coping strategy was focusing on and venting emotions. Some patients experienced fear-related thoughts and feelings prior to an activity: “We were going to do some tough hikes. At the first one I was like, ‘I will not be able to do this.’ And that just gave me way too much stress. I was also like ‘I want to go home. I’m not going to pull this off.’ In the end, I was able to do it easily” (P6). However, some patients also regularly experienced anger or irritation when activities cannot be performed as desired: “Then I couldn’t get a thread in the needle of a sewing machine either and then you start getting annoyed” (P3). Some patients regularly experienced sadness when activities cannot be performed as desired: “With my family we go out once a year. Then they have to think of what we can do and then there are many things I can’t do That gets me very sad on a regular basis” (P11).

Several patients mentioned that they regularly suppress competing activities to cope with their symptoms: “What I do regret is that I used to love going rock and roll dancing with my wife. That’s completely gone, and I think that’s a shame. … I can’t keep up the pace anymore” (P8). Contrary, some patients indicated that they often apply a restraint coping strategy: “If I want to do something, I’ll see how it goes. I’m not going to cancel in advance” (P4).

Some patients indicated applying behavioral or mental disengagement strategies. An example of behavioral disengagement, which includes reducing attempts to deal with symptoms and possibly giving up on achieving goals, is: “It’s easier to suffer pain than to face it” (P8). Mental disengagement includes performing activities to distract from the symptoms: “I don’t want to cancel. I’d rather go somewhere in pain. Distraction is good. It distracts from the pain, so when I’m doing other things, working on something, I realize less that I have this pain” (P7).

Finally, some patients tend to deny their situation. An example is: “I always say ‘I’m not sick’. … Sick people lie in bed, and they have fever. I don’t have all that. I have cancer. That’s something else. And neuropathy is a result of my treatment, but I’m not sick” (P8).

### Self-management: active

An active behavioral strategy that was mentioned by many patients is exercise. Low-to-moderate intensity exercises were specially mentioned, such as walking, cycling, pilates, and yoga: “I take a walk in the evening. I started doing pilates again because that also gives me some space” (P3) and “I have that bike seat and of course that helps. You keep your muscles warm and I’m not very stiff because of that” (P10). Patients frequently look for other ways to use objects to perform an action: “I did get some tools from an occupational therapist that make things like cutting and opening bottles easier” (P12). Some patients perform actions cautiously (“I walk down the stairs very strangely. I am so insecure about the ground and my feet. I hold on [to the banister] and always put my foot in. Preferably still at an angle so you can put your whole foot on the step” (P4)). Furthermore, some patients mentioned that they use work or social activities to self-manage symptoms: “Sometimes I get up in the morning and I think ‘oh, I am so tired’ and then I have to go to work. If I sit there and I have that distraction then I’m fine” (P1).

### Self-management: cognitive (active)

Few patients reported using cognitive (active) self-management strategies. One patient indicated keeping a diary: “Then I started writing diaries. And that helps, too. On average, I do that 2-3 times a week. Sometimes the nice things, sometimes the not so nice things” (P11). Another patient indicated performing mindfulness exercises: “I once did mindfulness training provided by the hospital. I still do use that occasionally” (P3).

### Self-management: passive behavioral

Making conscious choices regarding wearing shoes was a passive behavioral self-management strategy named by many patients, which varied widely from barefoot (“I always walk barefoot at home now. Then I feel the contact with the ground.” (P2)) to hiking boots (“From that moment I had pain in the feet and then I started wearing high shoes. Nowadays I always walk on walking shoes” (P11)). Furthermore, many patients indicated they need rest to recover: “Sometimes I say ‘no I’m not going’. I have to cancel. I don’t feel well enough. Then I usually stay home and sleep or rest a little more” (P5). Several patients also indicated that they often sit down during activities to relieve symptoms: “Taking breaks with cooking is a little more difficult, of course, but then I just sit down with cutting or things like that” (P4). Some patients also report that keeping their hands or feet warm to relieve their symptoms: “So if it is very cold outside later, I will have to dress extra for that” (P9). In addition, some patients indicated that they benefit from adjustments in bed, wearing gloves, keeping feet/legs in a certain position, wearing socks, and taking a bath or shower. Some patients also reported drinking alcohol to reduce their symptoms: “If you have very sore feet and you walk on anyway, I sleep badly because then those feet still hurt at night. Then I usually have a glass of wine” (P1). Furthermore, some patients look for alternatives in transportation (“I can’t drive for long because my feet will protest. I go by train or I go on the electric bike” (P4)).

### Self-management: conventional medical

Many patients use or have used medication to control their symptoms. This differs between over-the-counter medication (“I take paracetamol very often. Still often 3 times 2 a day” (P4) and prescription medication (“If I haven’t been able to sleep for a few nights because my leg hurts bad, I take medication, because you do want to sleep. … Then you take paracetamol or naproxen” (P10)). In terms of healthcare professionals, the physical therapist is the most consulted type to control symptoms: “With the physical therapist, I had to start doing balance exercises. Sometimes you get up and then I couldn’t find my balance” (P6). Other resources named by a few patients included orthotics, psychologists, supplements, general practitioners, physicians, occupational therapists, osteopaths, life art coaches, coaches, and a rollator walker.

## Discussion

This study qualitatively examined coping (i.e., dealing with painful neuropathic sensations and resulting impairments in general) and self-management (i.e., practical actions to reduce symptoms) strategies that were perceived as helpful by patients with chronic painful CIPN. Whereas patients are often told that they simply have to learn to live with CIPN, this study has shown that patients experience it as a difficult road in which they employ various coping and self-management strategies. Strategies that many patients employ are, for example, planning, acceptance, and suppressing competing activities (i.e., coping), as well as practical actions such as medication and sitting (i.e., self-management).

This study has shown that in addition to many patients employing active coping strategies (e.g., planning and acceptance) that are generally considered effective, a lot of patients also often apply regulation strategies that are generally considered non-helping and aversive in the long term as has also been shown in research [32, 44, 45]. Examples of such strategies are focus on and venting of emotions, suppression of competing activities, and mental disengagement. According to the approach-avoidance coping model, these strategies are part of avoidance coping, meaning that patients try to ignore, avoid, or withdraw from the stressor (i.e., painful neuropathic sensations and resulting impairments) [46, 47]. Approach coping, on the other hand, includes patients trying to actively reduce, manage, or eliminate the stressor [46, 47]. Biopsychosocial research has shown that avoidance strategies can work in the short term but lead to worsened outcomes in the long term (e.g., pain and disability) [46–49]. Because approach coping strategies are more effective in the long run [46–50], patients should be encouraged to adopt associated strategies of which relatively few or no patients in our study indicated they have adopted, such as acceptance, seeking support, and positive reinterpretation. Psychosocial interventions such as cognitive behavioral therapy (CBT) may help patients adopt helping strategies such as improving planning or reducing focusing on and venting emotions [17]. Other psychosocial interventions such as acceptance and commitment therapy (ACT) can help patients counteract experiential avoidance. The mechanism underlying ACT can be explained through relational frame theory [51]. Because patients have associations and make connections through language, experiencing limitations due to CIPN can cause people to feel less worthy of themselves or to want to avoid associated emotions. This is also due to the association that CIPN has with cancer. ACT focuses on this mechanism and helps patients to become more psychologically flexible [52] allowing them to better cope with their symptoms. Future directions in research should focus on developing suitable psychosocial interventions for patients with chronic painful CIPN to support them in managing their symptoms.

A wide variety of self-management strategies emerged in this study, with many patients indicating that exercise helped them reducing, managing, or eliminating their neuropathic sensations. Most recent ASCO (American Society of Clinical Oncology) recommendations do not include exercise, but it does indicate that recent preliminary research shows the potential benefits of exercise [21]. However, recent ESMO (European Society for Medical Oncology) recommendations do include exercise as the amount of evidence is growing [20]. Physical exercise and functional training are recommended as these are shown to reduce CIPN symptoms. It improves physical functioning, which can prevent further deterioration of impairments and falls [20]. Additionally, a recent systematic review into the treatment of CIPN concluded that some studies reported that exercise shows short-term improvements in CIPN symptoms, such as pain intensity, physical functioning, and balance, but that statistical and clinical significance varied among studies [53]. Another recent systematic review named that effects appear positive, but studies have low sample sizes and were heterogeneous regarding the used methodology [54]. In conclusion, it seems that exercise is generally recommended for patients with CIPN, but more research on this is needed to demonstrate evident effectiveness [20, 21, 54, 55]. Possibly improved physical state and interference may also positively affect patients’ sense of control in life and therefore the way they deal with their symptoms. Therefore, future research should also examine whether improvements in physical functioning might influence how people cope with their symptoms.

Furthermore, many patients indicated that they approached different healthcare professionals for support in dealing with CIPN. Physical therapy was mostly mentioned by patients in our study in terms of healthcare professionals. There are currently limited recommendations regarding physical therapy [20, 21]. Further referral to physical therapists is included for support in mainly sensory impairment of CIPN [20]. Advice is mainly practical, namely in ADL support. Recommendations relate to dressing (e.g., extension loops on zippers for opening zippers) and body hygiene (e.g., electric toothbrush). Advice is also given for housekeeping (e.g., slip-proof handles) and work (e.g., work settings). It is also mentioned that advice can always be discussed with occupational therapists or physical therapists for additional guidance [20]. However, little research has been done on broader approaches such as rehabilitation, which often combines the above elements. As patients may experience impaired mobility and therefore an increased risk of falling, appropriate rehabilitation for functional limitations is needed to maintain QoL [56]. A review that examined the treatment of CIPN and the implementation of physical therapy and rehabilitation has developed recommendations for exercise and rehabilitation protocols [57]. These recommendations include all patients with CIPN following home-based exercise programs, with low-to-moderate intensity progressive walking and resistance exercises, to reduce or eliminate CIPN symptoms. Furthermore, it is recommended that patients with more severe CIPN symptoms, such as impaired balance and coordination, trouble walking, recurrent falls, pain, severe numbness and tingling, impaired mobility, and durable medical equipment needs, attend an outpatient rehabilitation program. Inpatient rehabilitation is described as crucial when patients have additional rehabilitative, medical, or rehabilitation nursing needs [57]. However, current ASCO and ESMO recommendations do not include rehabilitation [20, 21]. Healthcare professionals should assess individual patients’ physical functioning problems and need to refer them on time to healthcare professionals such as physiotherapists, occupational therapists, or rehabilitation teams, to limit problems in physical functioning such as disability and falls [56, 58].

### Strengths and limitations

Several strengths of this study can be mentioned. As far as we are concerned, where previous studies looked at either coping or self-management strategies, this is the first study that examined both coping and self-management strategies in patients with chronic painful CIPN. Furthermore, interviews were conducted online via video calling. This makes participation in the study more accessible, enabling vulnerable and disabled patients to sign up for participation as well. This might have contributed to a representative sample. Furthermore, this study used a hybrid inductive-deductive thematic analysis. As a result, all patients’ coping and self-management strategies were included in the results in a structured way.

This study also has some limitations. First, patients were only recruited through online flyers via patient organizations and *Kanker.nl*, which may have resulted in a biased sample. Second, the sample of this study mainly involves patients who have suffered from CIPN for more than 5 years. Earlier research on other chronic conditions, such as rheumatoid arthritis, low back pain, and chronic fatigue syndrome, has shown that patients with higher symptom duration report different coping strategies than those with lower symptom duration [59–61]. As a result, this study may provide less insight into patients who have only had CIPN for a shorter period and have yet to learn to live with their symptoms. Third, most patients in this sample are older than 60 years, so young cancer patients are underrepresented. Previous research has shown that younger adults appear to exhibit different coping strategies than older adults due to both the personal development of coping mechanisms and the influence of environmental factors that vary by age and stage of life [62]. In addition, older age appears to be associated with fewer coping strategies [62]. However, since the majority of the population of cancer survivors is over the age of 60, the results can still be considered representative of a large proportion of patients. Fourth, this study described only the self-management strategies that patients found helpful. However, patients also named many non-helpful self-management strategies that they had tried in the past. These were not described in this study as this study focused on the helpful strategies that people use to deal with the symptoms. It is possible that these unmentioned strategies could be valuable to other patients, but they are not represented in this article. Nor have we now gathered insight into the strategies that were ineffective or even counterproductive from the patients’ perceptions. Fifth, no intercoder reliability was considered in this study because there was only one coder. This may potentially have affected the validity of the codes and themes that emerged from the data [63].

## Conclusion

This study aimed to identify helpful coping and self-management strategies of patients with painful chronic CIPN. Coping and self-management strategies that patients employ diverge greatly. Future directions in research should investigate psychosocial interventions to support patients to adopt helpful coping strategies. Furthermore, healthcare professionals need to monitor symptoms to refer patients to appropriate healthcare promptly when needed to limit the deterioration of physical state and functioning.

## Data Availability

The datasets generated during and/or analyzed during the current study are available from the corresponding author on reasonable request.
